# Impact of sleep duration, physical activity, and screen time on health-related quality of life in children and adolescents

**DOI:** 10.1186/s12955-021-01776-y

**Published:** 2021-05-12

**Authors:** Carlos K. H. Wong, Rosa S. Wong, Jason P. Y. Cheung, Keith T. S. Tung, Jason C. S. Yam, Michael Rich, King-Wa Fu, Prudence W. H. Cheung, Nan Luo, Chi Ho Au, Ada Zhang, Wilfred H. S. Wong, Jiang Fan, Cindy L. K. Lam, Patrick Ip

**Affiliations:** 1 Department of Family Medicine and Primary Care, LKS Faculty of Medicine, The University of Hong Kong, Hong Kong, SAR China; 2 Department of Pharmacology and Pharmacy, LKS Faculty of Medicine, The University of Hong Kong, Hong Kong, SAR China; 3 Department of Paediatrics and Adolescent Medicine, LKS Faculty of Medicine, The University of Hong Kong, Room 115, 1/F, New Clinical Building, 102 Pokfulam Road, Queen Mary Hospital, Hong Kong, SAR China; 4 Department of Orthopaedics and Traumatology, LKS Faculty of Medicine, The University of Hong Kong, Hong Kong, SAR China; 5 Department of Ophthalmology and Visual Sciences, Faculty of Medicine, The Chinese University of Hong Kong, Hong Kong, SAR China; 6 Center on Media and Child Health, BCH3186, 300 Longwood Avenue, Boston, MA 02115 USA; 7 Journalism and Media Studies Centre, The University of Hong Kong, Hong Kong, China; 8 Saw Swee Hock School of Public Health, National University of Singapore, Singapore, Singapore; 9 Department of Developmental and Behavioral Pediatrics, Shanghai Children’s Medical Center, Shanghai Jiao Tong University School of Medicine, Shanghai, China; 10 Ministry of Education-Shanghai Key Laboratory of Children’s Environmental Health, Shanghai, China

**Keywords:** Screen time, Physical activity, Sleep duration, Health-related quality of life, Adolescents, Children

## Abstract

**Background:**

Existing studies on health-related quality of life (HRQoL) mainly covered single growth stages of childhood or adolescence and did not report on the trends in the relationships of HRQoL with sleep duration, physical activity, and screen time. This study aimed to establish the population norm of HRQoL in children and adolescents aged 6–17 years and examine the associations of screen time, sleep duration, and physical activity with HRQoL in this population.

**Methods:**

We conducted a large-scale cross-sectional population-based survey study of Hong Kong children and adolescents aged 6 to 17 years. A representative sample of students were interviewed to assess their HRQoL using PedsQL and EQ-5D-Y-5L. Multivariable homoscedastic Tobit regression with linear form or restricted cubic spline of predictors was used to analyze the associations between screen time, sleep duration, and HRQoL. Multiple imputation by chained equations was performed to deal with missing data.

**Results:**

A total of 7555 respondents (mean age 11.5, SD 3.2; 55.1% female) were sampled. Their EQ VAS scores, PedsQL physical summary scores, and psychosocial summary scores were positively correlated with sleep duration and moderate/vigorous activity but was negatively correlated with screen time.

**Conclusions:**

Children and adolescents who had longer exposure to screen, shorter sleep duration, and lower physical activity levels appeared to have poorer HRQoL as assessed by PedsQL and EQ-5D-Y-5L. Advice and guidance on screen time allocation for children and adolescents should be provided at the levels of school, community, and family.

## Background

Health-related quality of life (HRQoL) is an important multidimensional concept that includes emotional, physical, and social aspects of one’s life, and together with traditional definitions of health status enables doctors and researchers to quantify an individual’s perceived functioning [1, 2]. In recent years, HRQoL in children and adolescents has emerged to be an important health outcome, as it can measure the risk of precursors of disease and indicate the health status of the next generation [3]. However, childhood HRQoL has not been adequately studied, and most of the existing research have been conducted on children and adolescents with chronic diseases [4]. More research on HRQoL in children needs to be conducted to provide a thorough understanding of children’s health status [5].

A previous study in children and adolescents have shown that sleep duration, screen time, and physical activity level were correlated with HRQoL, and those with lower screen time and moderate physical activity/sedentary behavior had the greatest HRQoL [6]. A systematic review found a higher physical activity level was associated with a better HRQoL [4]. In analyzing adolescent’s 24-h time-use patterns, those with the highest physical activity and moderate screen time had the highest HRQoL [3, 7]. In children and adolescents, time allocation appears to be an important component affecting HRQoL [4, 6]. Moreover, compared with physical activity or sedentary behavior, stronger associations were found between health utility and sleep patterns [8]. In particular, use of screen-based media devices during one hour before bedtime was associated with a lower HRQoL in children and adolescents [9], suggesting that maintaining healthy sleep habits and reducing screen time is very important in children and adolescents.

In general, these studies have drawn our attention to the influence of sleep duration, screen time, and physical activity on health outcomes as measured by HRQoL [2–4, 6, 8, 9]. To the best of our knowledge, this topic has not been adequately studied in children. Only a few cross-sectional studies have analyzed sleep duration, screen time, and physical activity in association with HRQoL of children and adolescents [7, 10]. However, these studies mainly covered single growth stages of childhood or adolescence and did not report on the trends in the relationships of HRQoL with sleep duration, physical activity, and screen time. To address this research gap, our representative school-based survey aimed to study the population norm of HRQoL in children and adolescents aged 6–17 years, and to examine the associations of screen time, sleep duration, and physical activity with HRQoL.

## Methods

### Study sample

The study recruited a representative sample of children and adolescents from 12 primary and 7 secondary schools in Hong Kong. Schools in 5 districts (Hong Kong Island, Kowloon West, Kowloon East, New Territories West, and New Territories East) of Hong Kong were invited by stratified random sampling. For those schools who accepted the invitation, their students were then recruited and given the five-level version of EQ-5D-Y (EQ-5D-Y-5L) questionnaire and the Pediatric Quality of Life Inventory™ (PedsQL) questionnaire. The inclusion criteria for the study were students who (1) were aged between 6 and 17 years and studying at primary or secondary school at the time of the interview, (2) provided gender and date of birth, (3) completed the EQ-5D-Y-5L and PedsQL questionnaires, and (4) provided their parents/guardians’ written consent for their participation in this study.

Among the 8,225 respondents, 670 were excluded: 513 had missing data of either gender or date of birth, 68 reached the age of 18, and 89 did not complete the five items of the EQ-5D-Y-5L questionnaire. The final sample included 7,555 respondents, giving a data completion rate of 91.9%.

Table 1 shows the characteristics of respondents. The mean age was 11.5 years (SD 3.2) and over half of participants were female (55.1%). The proportions of respondents were 14.5%, 25.2%, 32.8%, and 27.4% across the monthly household income bands of less than $12,000, between $12,000 and $19,999, between $20,000 and $39,999, and over $40,000, respectively. The majority of parents (86.4%) were married, and 70.2% of mothers and 68.4% of fathers had a secondary school education.

**Table 1:** Descriptive statistics of participants by age groups

Characteristics	Total (N = 7555)	Age 6–8 (N = 1925)	Age 9–11 (N = 2535)	Age 12–17 (N = 3095)	*P*-value
Age, mean (SD)	11.5 (3.2)	7.7 (0.7)	10.5 (0.8)	14.8 (1.7)	< 0.001*
*Gender, % (n)*					< 0.001*
Female	55.1% (4166)	52.1% (1003)	52.3% (1325)	59.4% (1838)	
Male	44.9% (3389)	47.9% (922)	47.7% (1210)	40.6% (1257)	
EQ-VAS, mean (SD)	82.7 (18.5)	88.9 (18.7)	85.2 (17.6)	77.1 (17.5)	< 0.001*
*PedsQL, mean (SD)*					
Physical functioning	85.1 (14.1)	86.1 (15.5)	88.1 (12.5)	82.2 (13.8)	< 0.001*
Emotional functioning	72.9 (19.9)	76.3 (20.7)	75.6 (19.2)	68.7 (19.2)	< 0.001*
Social functioning	84.4 (17.0)	85.2 (18.3)	86.8 (16.0)	82.0 (16.6)	< 0.001*
School functioning	78.4 (16.5)	80.9 (17.4)	82.2 (14.9)	73.7 (16.0)	< 0.001*
Psychosocial health summary score	78.6 (14.9)	80.8 (15.7)	81.5 (13.9)	74.8 (14.3)	< 0.001*
Physical health summary score	85.1 (14.1)	86.1 (15.5)	88.1 (12.5)	82.2 (13.8)	< 0.001*
Total score	80.9 (13.4)	82.6 (14.3)	83.8 (12.3)	77.4 (12.8)	< 0.001*
*IPAQ, mean (SD)*					
Sedentary activities, min per day	312.7 (223.0)	224.5 (483.3)	287.9 (354.4)	388.0 (320.0)	< 0.001*
Walking, min per day	73.3 (72.3)	60.3 (137.7)	82.1 (140.6)	74.2 (105.4)	< 0.001*
Moderate activities, min per day	41.4 (42.5)	37.2 (120.8)	45.9 (87.5)	40.3 (95.5)	0.002*
Vigorous activities, min per day	32.3 (36.6)	24.9 (120.4)	35.5 (77.8)	34.3 (80.0)	< 0.001*
Total physical MET score	4662.4 (3325.9)	3827.3 (8459.6)	5173.3 (7278.7)	4764.0 (6906.3)	< 0.001*
*IPAQ Physical level, % (n)*					< 0.001*
Low	2.6% (198)	4.0% (76)	2.7% (52)	1.7% (33)	
Moderate	29.9% (2261)	38.0% (962)	26.0% (658)	28.2% (715)	
High	67.4% (5096)	58.1% (1796)	71.4% (2207)	70.1% (2168)	
*Use of electronic devices, hours per day, mean (SD)*					
Weekdays					
Screen time for studying	1.0 (1.2)	0.7 (2.1)	0.9 (2.1)	1.3 (2.0)	< 0.001*
Screen time for gaming and leisure	3.5 (3.4)	2.3 (5.2)	2.8 (5.3)	4.8 (6.0)	< 0.001*
Total screen time	4.5 (3.7)	3.0 (6.2)	3.7 (5.7)	6.0 (6.2)	< 0.001*
Weekends					
Screen time for studying	1.1 (1.3)	0.8 (2.4)	1.0 (2.4)	1.3 (2.4)	< 0.001*
Screen time for gaming and leisure	6.4 (4.3)	5.1 (7.9)	5.9 (8.3)	7.6 (6.4)	< 0.001*
Total screen time	7.5 (4.4)	5.9 (8.6)	6.9 (8.2)	8.9 (6.4)	< 0.001*
*Overall*					
Screen time for studying	1.0 (1.1)	0.7 (2.0)	0.9 (2.1)	1.3 (2.0)	< 0.001*
Screen time for gaming and leisure	4.3 (3.4)	3.1 (5.6)	3.7 (5.7)	5.6 (5.7)	< 0.001*
Total screen time	5.3 (3.6)	3.9 (6.5)	4.6 (5.9)	6.8 (5.9)	< 0.001*
*Sleeping duration, hours, mean (SD)*					
Weekdays	8.3 (1.4)	9.1 (2.5)	8.8 (1.7)	7.5 (2.0)	< 0.001*
Weekends	9.5 (1.5)	9.9 (2.1)	9.7 (2.8)	9.0 (2.4)	< 0.001*
Overall	8.6 (1.2)	9.3 (2.0)	9.0 (1.6)	7.9 (1.8)	< 0.001*
*Household monthly income, % (n)*					< 0.001*
< HK$12,000	14.5% (588)	12.7% (138)	15.4% (217)	15.0% (233)	
HK$12,000–$19,999	25.2% (1021)	23.5% (256)	24.5% (344)	27.1% (421)	
HK$20,000–$39,999	32.8% (1330)	32.2% (351)	29.6% (417)	36.2% (562)	
≥ HK$40,000	27.4% (1111)	31.6% (344)	30.5% (429)	21.8% (338)	
*Parental characteristics, % (n)*					
Marital status					< 0.001*
Married	86.4% (3529)	90.5% (994)	87.5% (1241)	82.6% (1294)	
Divorced/Separated	11.9% (486)	7.3% (80)	11.1% (157)	15.9% (249)	
Cohabitation	1.7% (70)	2.3% (25)	1.5% (21)	1.5% (24)	
Education level					
Mother					< 0.001*
Primary school or below	7.5% (310)	3.6% (40)	6.6% (94)	11.1% (176)	
Secondary school	70.2% (2899)	65.0% (723)	67.7% (968)	76.0% (1208)	
Tertiary/University or above	22.3% (921)	31.4% (349)	25.7% (367)	12.9% (205)	
*Father*					< 0.001*
Primary school or below	5.9% (238)	2.8% (31)	4.7% (66)	9.0% (141)	
Secondary school	68.4% (2780)	63.7% (695)	65.4% (920)	74.5% (1165)	
Tertiary/University or above	25.7% (1044)	33.5% (365)	29.9% (421)	16.5% (258)	

IPAQ, International Physical Activity Questionnaire; MET, Metabolic equivalent of task; SD, standard deviation

*Significant difference (*p* < 0.05) between groups by univariate linear regression, or multinomial logistic regression, as appropriate

### Data collection

The survey had three versions designed for primary school students in grade 1–3 and grade 4–6, and secondary school students, respectively. Survey hardcopies were delivered to schools, and teachers distributed the hardcopies to each student. Both self-reported and proxy-reported questionnaires were applied according to the age group. EQ-5D-Y-5L and PedsQL questionnaires were read and answered by the students themselves in class. Teachers were available to answer students’ questions on the survey. For the demographic, IPAQ, sleep duration and screen time questionnaires, parents of primary students in Grade 1–3 were instructed to complete them at home and return them to schools upon completion; for older students, they completed the questionnaires by themselves in class.

### Study instruments

#### EQ-5D-Y-5L

The EQ-5D-Y is the youth version of EQ-5D and is designed as a child-specific and age-appropriate measure of HRQoL [11]. The instrument consists of five dimensions: “mobility”, “looking after myself”, “usual activities”, “pain/discomfort”, and “feeling worried/sad/unhappy”. It has been shown to be valid and reliable in assessing HRQoL in children aged 8 to 15, but should be used with a degree of caution in healthy children and children with chronic health conditions [11, 12]. The EQ-5D-Y is short questionnaire that is responsive to changes and is generally acceptable, especially in acutely ill children. It has been used to evaluate HRQoL in children and adolescents with various diseases [13, 14]. The EQ-5D-Y-5L, a five-level version of the EQ-5D-Y, has been demonstrated to be responsive and suitable for certain diseases among the young population in precedent studies [15, 16].

##### PedsQL

The PedsQL instrument is a modular approach for measuring HRQoL in healthy children and adolescents, and those with acute and chronic health conditions. The PedsQL 4.0 Generic Core Scales contains 23 items divided into four scales: physical functioning (8 items), emotional functioning (5 items), social functioning (5 items), and school functioning (5 items). It has three summary scores including total score, physical health summary score, and psychosocial health summary score. The reliability and validity of PedsQL have been tested in a previous study [17].

#### IPAQ

The physical activity level of students was measured using the International Physical Activity Questionnaire (IPAQ), which was completed by the students themselves unless they were in grade 1–3 in primary school [18]. It can assess sedentary activities, walking, moderate activities, vigorous activities, and total metabolic equivalent task (MET) scores.

### Screen time and sleep duration

Respondents were asked about their usage of electronic devices and their sleep duration on weekdays and weekends (hours per day). The use of electronic devices with screens (e.g., smart phones, computers, tablets, etc.) was assessed by the amount of screen time spent studying and the amount of screen time spent on gaming and leisure. Average daily screen time and average sleep duration on weekdays and at weekends were calculated based on the weighted average on weekdays and at weekends as (weekday * 5 + weekend * 2)/7.

### Statistical analysis

Durations of screen time, walking/moderate/vigorous activities and sleep were regarded as outliers if their total sum was larger than 24 h per day. The components of these sums were excluded and estimated using multiple imputation. Total sums larger than 24 h per day after multiple imputation were rescaled to 24 h per day based on the individual proportions in the sum.

Restricted cubic spline regression analyses were used for modeling linear/non-linear relationships between the different durations and HRQoL on a continuous scale [19]. Relevant variables with completion rate larger than 50% were included in the models for adjustment. These included age, sex, schools, household income, and parental information, including marital status and education levels. The four outcomes were EQ-VAS, physical health summary score, psychosocial health summary score, and total PedsQL score. As the four outcomes were measured from 0 to 100, multivariable homoscedastic Tobit models were used to account for ceiling and floor effects [20]. As the sample size is larger than 100, five knots at 5th, 27.5th, 50th, 72.5th, and 95th percentiles of the distribution were used to model durations of moderate or vigorous physical activity, screen time, and sleep duration in the restricted cubic spline analyses. Separate restricted cubic spline regressions were conducted in the four outcomes. Likelihood ratio (LR) tests were used to determine whether a Tobit model with restricted cubic spline of predictor provided better fit than a Tobit model with linear form of predictor. To visualize these relationships, predicted EQ-VAS was plotted for each duration, stratified by the three age groups (ages 6–8, 9–11, and 12–17) and by sex.

The distribution of responses and ceiling and floor effects of EQ-5D-Y-5L were reported. The PedsQL psychosocial health summary score, physical health summary score, and total score were also calculated. The p-trend test was conducted to show trends in the proportion of ‘no problems’ among different age groups.

Abnormal data (e.g. sleep time + screen time + walking/moderate/vigorous > 24 h per day) was excluded and the durations were rescaled. Incomplete data on the duration of physical activity, screen time, and sleeping were handled by multiple imputation by chained equations (MICE) [21]. Each missing duration datum was imputed five times in random chained equations by gender, age, and school. Five complete imputed datasets were generated and analyzed individually. Results of the model parameters were then combined into a single estimate by applying Rubin’s rules [22].

All statistical analyses were performed using STATA Version 16. Statistical significance was set at *p* < 0.05.

### Ethical approval

Approval was obtained from the Institutional Review Board of the University of Hong Kong—the Hospital Authority Hong Kong West Cluster (reference number: UW 18-139).

## Results

### EQ-5D-Y-5L

**Fig. 1:**
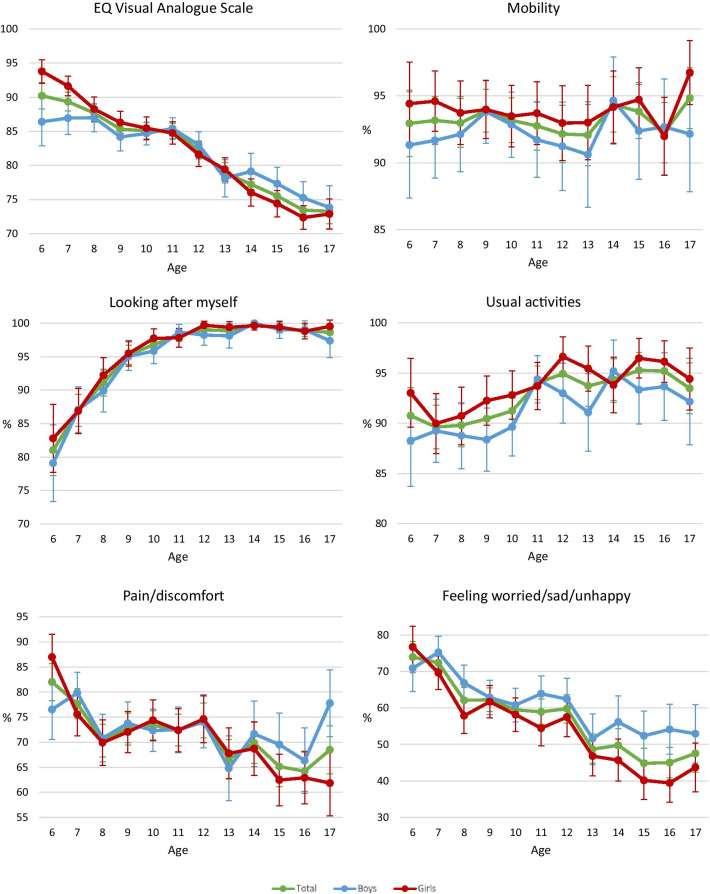
Trends in mean and 95% confidence interval of EQ-VAS and proportion of 'no problems' in the EQ-5D-Y-5L dimensions of mobility, looking after myself, usual activities, pain/discomfort, and feeling worried/sad/unhappy by sex and age

Table 2 shows that 93.2%, 95.3%, 92.5%, 71.7%, and 58.1% of respondents reported ‘no problems’ in the dimensions of mobility, looking after myself, usual activities, pain/discomfort, and feeling worried/sad/unhappy, respectively. Figure 1 displays the significant trends of the proportion of ‘no problems’ in looking after myself, usual activities, pain/discomfort, and feeling worried/sad/unhappy by sex (*p* < 0.001). Overall, the proportion of girls with ‘no problems’ in mobility and usual activities was higher than boys, whereas the proportion of girls with ‘no problems’ in feeling worried/sad/unhappy was lower than boys (Fig. 1).

**Table 2:** Distribution of EQ-5D-Y-5L dimension levels

EQ-5D-Y-5L dimension, % (n)	1	2	3	4	5	*P*-value
Mobility	93.2% (7038)	5.6% (426)	0.8% (64)	0.2% (16)	0.1% (11)	0.920
Looking after myself	95.3% (7200)	3.5% (263)	0.7% (50)	0.2% (18)	0.3% (24)	< 0.001*
Usual activities	92.5% (6986)	6.1% (461)	0.9% (69)	0.3% (21)	0.2% (18)	< 0.001*
Pain/discomfort	71.7% (5416)	24.3% (1833)	3.1% (232)	0.5% (41)	0.4% (33)	< 0.001*
Feeling worried/sad/unhappy	58.1% (4386)	31.2% (2360)	7.3% (548)	1.9% (146)	1.5% (115)	< 0.001*

*Significant difference (*p* < 0.05) between age groups by ordered logistic regression

The mean EQ-VAS score was 82.7. Specifically, the mean EQ-VAS score was 82.8 in boys and 82.6 in girls. There were significant differences in the EQ-VAS scores among the three age groups: age 6–8, age 9–11, and age 12–17 (mean 88.9, 85.2, 77.1, *p* < 0.001) and between primary school and secondary school categories (mean 86.6, 76.0, *p* < 0.001). Figure 1 shows a gradual decline in EQ-VAS scores from the age of 6 to 17 years (*p* < 0.001). Between the age of 6 and 8, the mean EQ-VAS score in girls was higher than in boys but lower after the age of 13.

### PedsQL

**Fig. 2:**
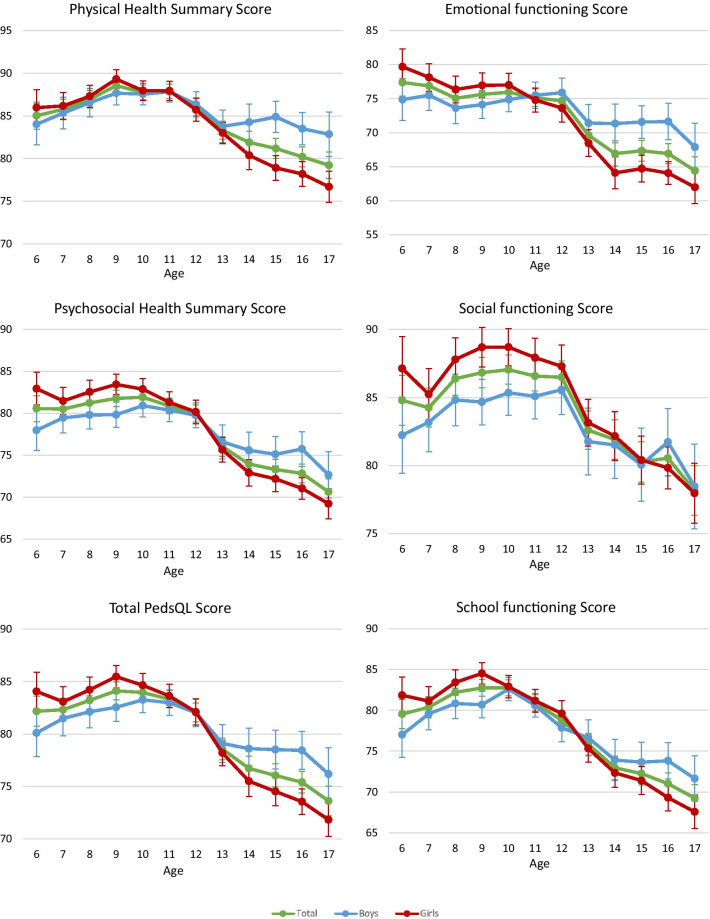
Trends in mean and 95% confidence interval of PedsQL emotional functioning score, social functioning score, school functioning score, psychosocial functioning score, physical functioning score, and total PedsQL score by sex and age

Table 1 shows the PedsQL psychosocial health summary scores (mean 78.6, SD 14.9; IQR 70.0–90.0), physical health summary scores (mean 85.1, SD 14.1; IQR 78.1–96.9), and total scores (mean 80.9, SD 13.4; IQR 72.8–91.3), respectively. Across the three scores, significant differences were observed among the three age groups (*p* < 0.001) and between primary and secondary schools (*p* < 0.001). Significant differences were also observed separately for boys and girls (*p* < 0.001) and the significant trend (*p* < 0.001) is shown in Fig. 2.

Emotional functioning scores and physical health summary scores in girls were slightly higher than in boys below 11 years of age, but were lower above 11 years of age. School functioning scores, psychosocial health summary scores, and total PedsQL scores in girls were higher than in boys below 12 years of age, but were lower above 12 years of age. Additionally, the social functioning score in girls was higher than in boys below 15 years of age (Fig. 2).

### IPAQ

The IPAQ scores (minutes per day) for sedentary activities, walking, moderate activities, and vigorous activities, and total MET scores were 312.7 (SD 223.0), 73.3 (SD 72.3), 41.4 (SD 42.5), 32.3 (SD 36.6), and 4662.4 (SD 3325.9), respectively. Overall, 67.4% of respondents had high physical activity levels, 29.9% had moderate physical activity levels, and 2.6% had low physical activity levels.

### Screen time and sleep duration

The mean screen time (hours per day) spent studying and on gaming/leisure on weekdays were 1.0 (SD 1.2) and 3.5 (SD 3.4), and at weekends were 1.1 (SD 1.3) and 6.4 (SD 4.3), respectively.

### Regression analysis

**Fig. 3:**
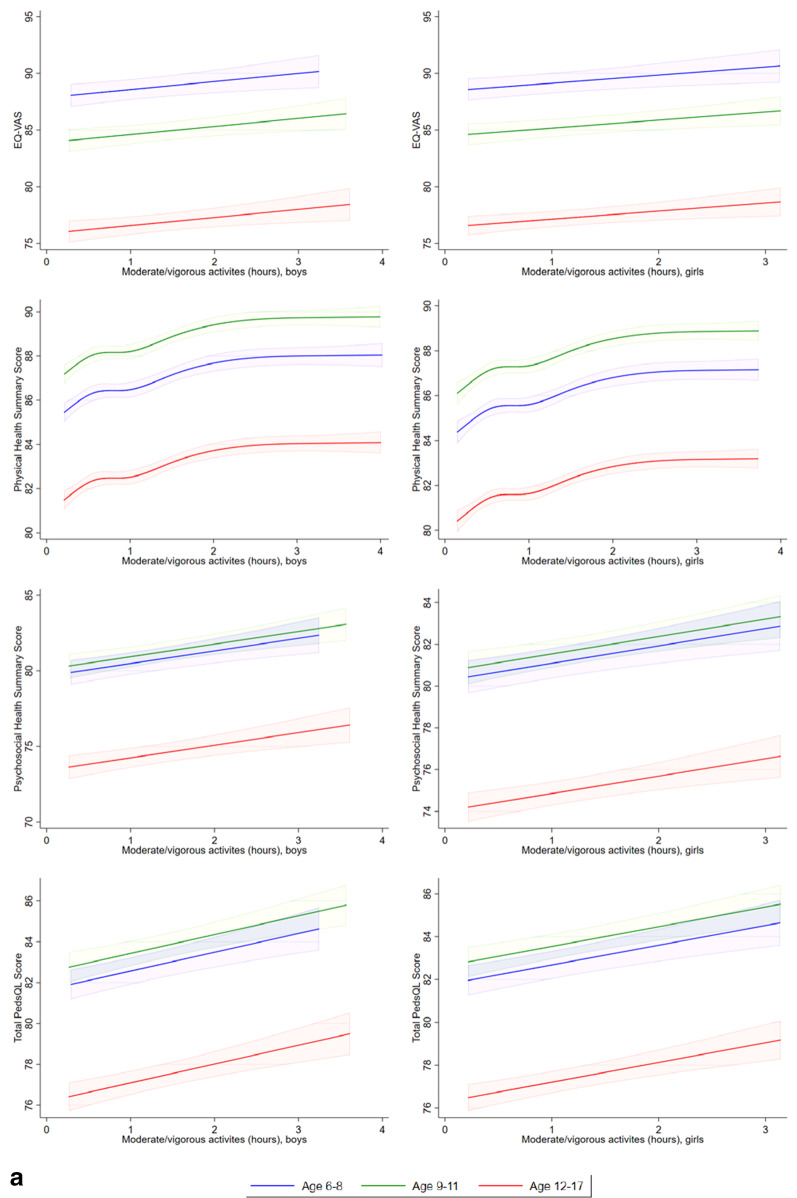

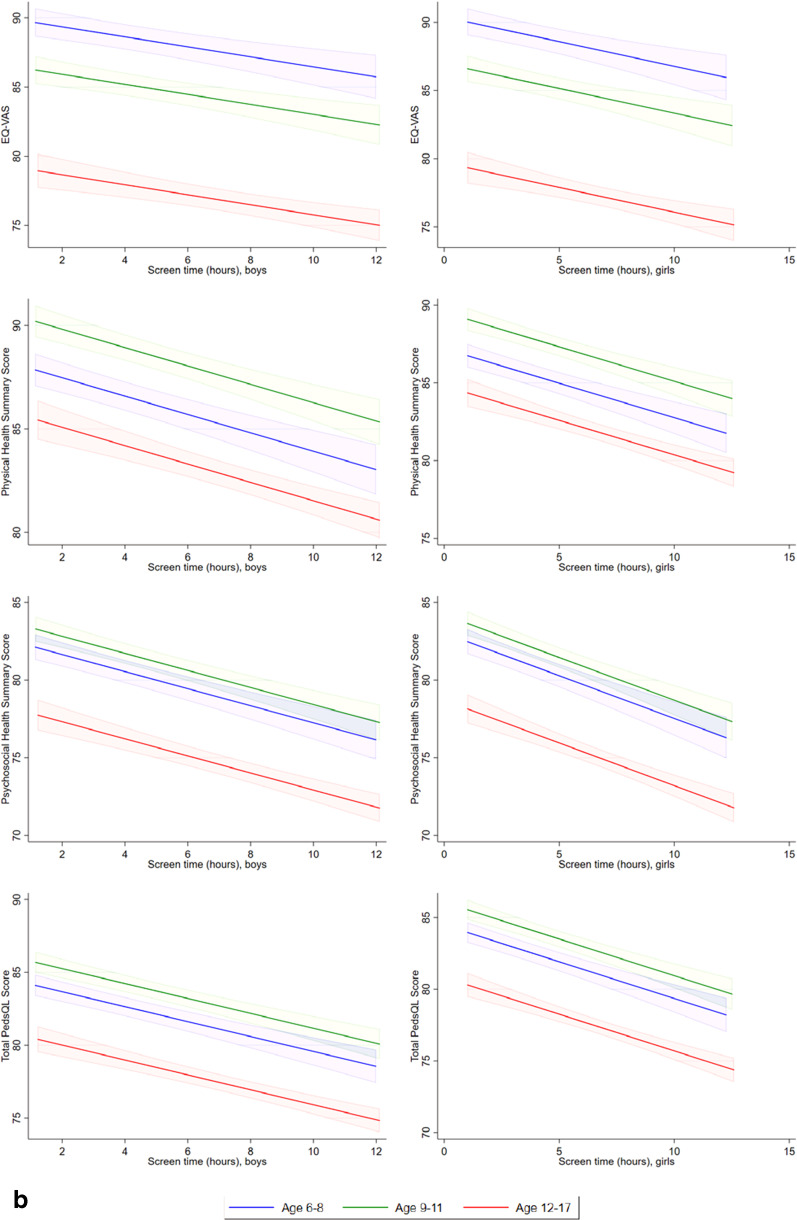

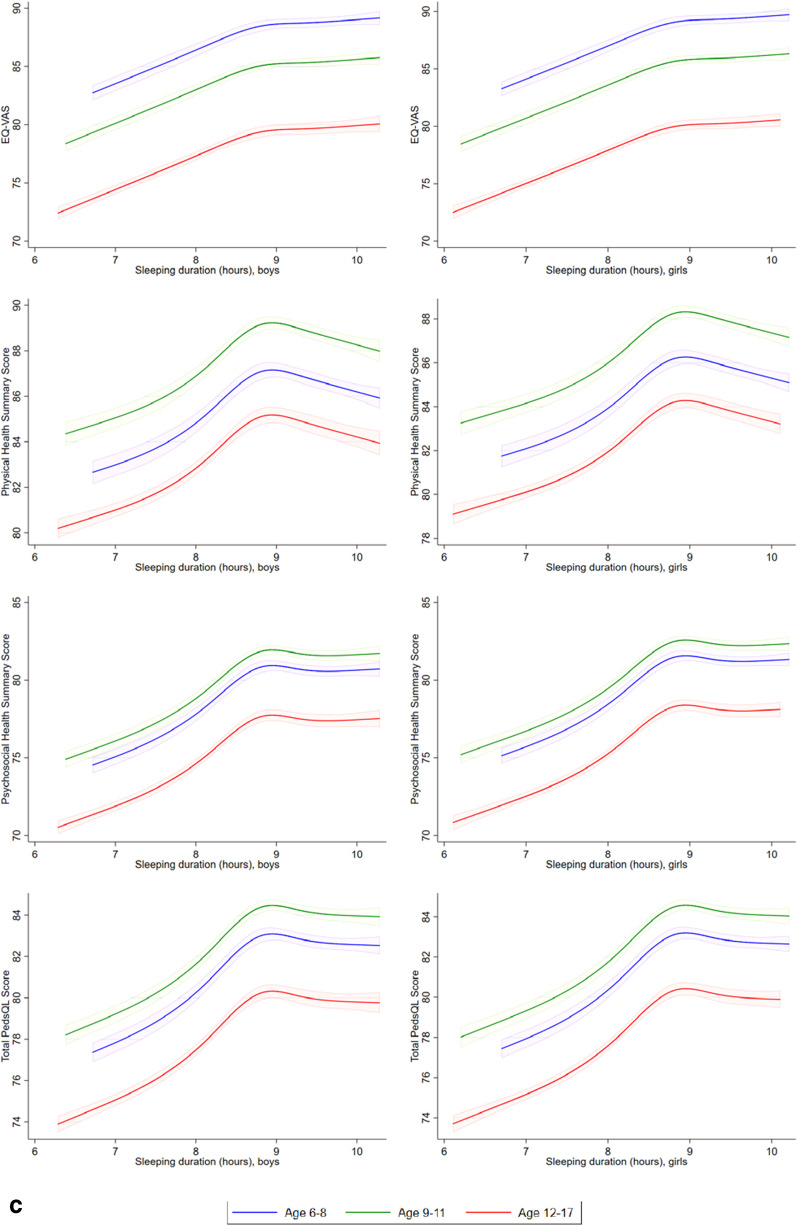
**a** Linear or restricted cubic spline plot of the association of moderate or vigorous physical activity (hours per day) with health-related quality of life measured by EQ-VAS, PedsQL physical health summary score, psychosocial health summary score, and total score, stratified by age and sex. **b** Linear plot of association of screen time for studying, gaming or leisure (hours per day) with health-related quality of life measured by EQ-VAS and PedsQL physical health summary, psychosocial health summary and total scores, stratified by age and sex. **c** Restricted cubic spline plot of association of sleeping duration (hours per day) with health-related quality of life measured by EQ-VAS and PedsQL physical health summary, psychosocial health summary and total scores, stratified by age and sex.

Comparing Tobit models with linear form and restricted cubic spline model, the LR test showed that the restricted cubic spline models provided better fit than linear model in the relationships between sleeping duration and EQ VAS scores, PedsQL physical summary scores, psychosocial summary scores, and total scores (LR = 22.5, *p* < 0.001, LR = 54.6, *p* < 0.001; LR = 47.5, *p* < 0.001; LR = 61.5, *p* < 0.001, respectively), screen time and EQ-VAS (LR = 8.6, p = 0.035), and time spent on moderate or vigorous activities and PedsQL physical summary scores (LR = 47.5, *p* < 0.001), indicating the fitting non-linear effects. Other relationships were estimated by Tobit models with linear form of predictors. Tobit models with linear form of predictors and Tobit models with restricted cubic spline of predictors were used to investigate the linear/nonlinear relationships between EQ-VAS score, PedsQL physical summary score, psychosocial summary score, total score, sleep duration, screen time, and moderate/ vigorous activity level. Figure 3a–c shows there were increases in EQ VAS scores, PedsQL physical summary scores, psychosocial summary scores, and total scores in children or adolescents with more time spent on moderate or vigorous activities, with less time spent using electronic devices, and with increasing sleep duration.

## Discussion

This study demonstrated associations between screen time, sleep duration, and physical activity level with HRQoL in children and adolescents, which is consistent with the previous findings. Moreover, the current study also used EQ-5D-Y-5L and PedsQL to measure the population norms of HRQoL in a representative sample of children and adolescents in Hong Kong.

Instruments such as EQ-5D-Y-5L and PedsQL can be used to estimate population norms or reference values of HRQoL in specific populations. These figures can enable comparisons between different populations and clinical groups to help researchers and health policymakers [23], whereas reference data can be used to compare patients with specific conditions and to assess the burden of certain diseases [11]. Normative comparisons of age, gender, or socio-economic status can identify subgroups that deviate from normal values to detect the impact of disease [24].

To the best of our knowledge, EQ-5D-Y-5L lacks a value set, which limits its use as a generic preference-based single index measure of benefit for use in cost-utility analysis [11]. Meanwhile, the population norms from PedsQL are not yet available anywhere in the world. In this study, using the EQ-5D-Y-5L and PedsQL, we demonstrated associations of screen time, sleep duration, and physical activity level with HRQoL in the general population of children and adolescents. On the other hand, the usefulness and reliability of EQ-5D-Y-5L and PedsQL in assessing HRQoL as an estimation of the health status of children and adolescents can be attested by examining these associations. Although the cubic spline regression analysis refuted the non-linear relationship hypothesis, the linear regression showed significant results, suggesting that the level of HRQoL among children and adolescents can be optimized by maximizing sleep and exercise duration and minimizing screen use duration.

The EQ-5D-Y-3L population norms in a Swedish study was comparable to the EQ-5D-Y-5L population norms in the current study. They found that girls reported more problems than boys in the EQ-5D-Y-3L dimensions of 'doing usual activities', 'having pain or discomfort' and 'feeling worried, sad or unhappy', and also lower mean VAS scores [25]. In general, our results were consistent with the Sweden study in the age range of 13 to 18 for VAS scores and for the dimensions of ‘pain/discomfort’ and ‘feeling worried/sad/unhappy’, but were inconsistent for ‘usual activities’ with more girls reporting ‘no problems’ in our study. In addition to the findings from the previous studies [3, 6–9]. our current study also examined HRQoL in children and adolescents by age and sex across a broad age range.

The current study found that children and adolescents with less screen time, more sufficient sleep, and higher physical activity level were generally associated with a greater HRQoL score, which is consistent with the findings from previous studies [3, 6–9]. However, majority of existing evidence pertain to the health impact of lifestyle patterns in a particular period such as childhood or adolescence. Very few studies have assessed the HRQoL of children and adolescents concurrently using the same self-reported instrument. Because of such high heterogeneity in measurement of HRQoL, it remains unclear as to what extent healthy lifestyle would benefit the well-being of children and adolescents. Our study thus extends previous research by covering a wider age range and demonstrates clearly a trend of HRQoL scores across ages for boys and girls in relation to their lifestyle patterns. Furthermore, the results inform future intervention design by highlighting the importance of adequate sleep and active living for enhancing HRQoL level during the critical developmental transition from childhood to adolescence.

The results in this study need to be interpreted with several caveats. First, this study adopted a cross-sectional design, which cannot be used to infer any causal relationship between screen time, sleep duration, and physical activity level on HRQoL. However, with the establishment of EQ-5D-Y-5L and PedsQL population norms for children and adolescents, future research should collect longitudinal data, through administration of these measures at multiple time points, to determine the temporal precedence between lifestyle habits and HRQoL. Second, this was an epidemiological survey study, which might be prone to volunteer reporting bias, as some subjects may be keen to join the study and report their data. Third, the outcome measures in this study were self-reported, and screen time, physical activity level, and sleep duration were not objectively measured. A well-designed cohort study with objective direct measurements such as use of wearable devices to collect real-time data on lifestyle patterns would help to clarify the relationships with HRQoL and provide more robust evidence to guide future policymaking.

## Conclusions

Time allocation in children and adolescents is highly associated with their HRQoL. Children and adolescents with more screen time, shorter sleep duration, and lower physical activity levels had lower HRQoL as measured by EQ-5D-Y-5L and PedsQL. Advice and guidance on screen time allocation in children and adolescents should be provided at the levels of school, community, and family.

## Data Availability

The datasets used and/or analysed during the current study are available from the corresponding author on reasonable request.

## Abbreviations

HRQoL: Health-related quality of life
IPAQ: International Physical Activity Questionnaire
PedsQL: Pediatric Quality of Life Inventory™
VAS: Visual Analogue Scale
SD: Standard deviation
MICE: Multiple imputation by chained equations
MET: Metabolic equivalent task
LR: Likelihood ratio
